# Intradentinal antimicrobial action and filling quality promoted by ultrasonic agitation of epoxy resin-based sealer in endodontic obturation

**DOI:** 10.1590/1678-7757-2017-0090

**Published:** 2017

**Authors:** Murilo Priori Alcalde, Clóvis Monteiro Bramante, Rodrigo Ricci Vivan, Pablo Andrés Amorso-Silva, Flaviana Bombarda de Andrade, Marco Antonio Hungaro Duarte

**Affiliations:** 1 Universidade de São Paulo, Faculdade de Odontologia de Bauru, Departamento de Dentística, Endodontia e Materiais Odontológicos, Bauru, SP, Brasil

**Keywords:** Endodontics, Bacteria, Ultrasonic

## Abstract

The aim of this study was evaluate the influence of ultrasonic activation (UA) of AH Plus to improve canal and isthmus filing, and analyse the antimicrobial effect against *Enterococcus faecalis* within dentinal tubules.

**Material and Methods:**

Thirty mesial roots of mandibular first molars were selected and divided into 2 groups (n = 15): with and without UA of the sealer. Then the root canals were filled by using the single cone technique, and the specimens were sectioned at 2, 4 and 6 mm from the apex for stereomicroscope and confocal laser scanner microscopy (CLSM) analysis. In addition, 30 bovine incisors were contaminated with *Enterococcus faecalis* and divided into 3 groups (n = 10). The specimens were obturated by using the single cone technique with (G1) and without (G2) UA of the sealer and G3 as the control group. All were sectioned into 6 mm-long cylinders and stained with LIVE/DEAD to assess bacterial viability by CLSM.

**Results:**

The UA of the sealer significantly reduced the presence of unfilled areas in the canal and isthmus area in all sections (p<0.05), and there was a significant increase in sealer penetration in both canals and isthmuses (p<0.05). As regards gaps, a significant reduction was found at 2 and 6 mm in the isthmus area of the UA group (p<0.05). Moreover, UA of the sealer significantly reduced bacterial viability in the superficial dentine when compared with the other groups (p<0.05).

**Conclusion:**

Ultrasonic activation of the AH Plus sealer promoted a better quality of root canal filling and increased the intratubular penetration of sealer, especially in the isthmus area. Additionally, ultrasonic activation of the sealer increased the intradentinal antimicrobial action against *Enterococcus faecalis,* mainly in the superficial dentine of the root canal.

## Introduction

Adequate obturation of the root canal system is required after biomechanical procedures, to ensure the long-term success of endodontic treatment^20^. During root canal obturation, an important objective of using endodontic sealers is to fill the spaces that cannot be reached by gutta-percha^6^. However, the anatomical complexities of the root canal system (isthmuses, fins and ramifications) may also interfere with complete obturation^2^.

The presence of isthmuses has a negative effect on the quality of root canal filling^13^ as the obturation materials barely reach and fill these recesses^4^ ^,^ ^5^. To overcome these drawbacks and improve the filling quality in terms of reducing unfilled areas and gaps, and promoting more effective penetration of sealer into dentinal tubules, recent studies have recommended the ultrasonic activation (UA) of sealers before obturation with gutta-percha^7^ ^,^ ^16^. However, this effect has only been studied in single rooted teeth with single canals, and no studies evaluating these aspects in teeth with anatomical complexities have been conducted so far.

Within the dentinal tubules, bacteria such as *Enterococcus faecalis (E. faecalis)* have capacity to penetrate deeply and offer resistance to the antimicrobial agents used in endodontics^29^. These bacteria are the microorganisms most frequently isolated from persistent endodontic infections^19^ ^,^ ^22^. Although the AH Plus sealer has been studied by several authors, and has shown good antimicrobial effect against *E. faecalis* ^18^ ^,^ ^28^, this factor might be dependent on the depth of penetration of the sealer. In addition, sealer penetration into the dentinal tubules should be considered beneficial because of its bacterial entombing action^10^ ^,^ ^15^. Based on the foregoing considerations, it would be interesting to assess the effect of ultrasonic activation of the sealer to enhance the antimicrobial activity against *E. faecalis* within the dentinal tubules.

Therefore, the aim of the present study was to evaluate the influence of ultrasonic activation of the AH Plus sealer to improve root canal and isthmus filling in mandibular molars, and analyse the antimicrobial effect against *E. faecalis* within the dentinal tubules. The null hypotheses tested were as follows:

Ultrasonic activation of the endodontic sealer would not improve the filling quality of the canal and isthmus.

There would be no difference in the adaptation of the sealer/dentine interface and intratubular penetration when ultrasonic activation was performed in the mesial roots of mandibular molars.

The ultrasonic activation would not improve the intradentinal antimicrobial activity of AH Plus against *E. faecalis.*

## Material and methods

### Root canal and isthmus filling quality

#### Sample Selection

The ethics committee approved this research (CEP 788274). The sample calculation was performed before the mechanical test by using the G*Power v3.1 for Mac (Heinrich Heine, University of Düsseldorf) by selecting the Wilcoxon- Mann-Whitney test of the *t*-test family. The alpha-type error of 0.05, a beta power of 0.95, and a ratio N2/N1 of 1 were also stipulated. The test showed a total of 8 samples for each group as the ideal size for noting significant differences. However, we used an additional 20% of instruments to compensate possible atypical values that might induce samples loss. To select mandibular molars with similar anatomical characteristics, 30 extracted mandibular first molars were anatomically paired by means of micro-computed tomography (micro-CT) SkyScan model 1174 (SkyScan, Kontich, Belgium) with an isotropic resolution of 16.82 μm. The images obtained of each specimen were reconstructed with Nrecon software (v.1.6.3 NRecon; Bruker-microCT, Belgium), providing images of the internal structures of axial sections of the canals in BMP format.

Based on the three dimensional models, anatomical pairing was achieved by selecting teeth of similar lengths and presenting the mesial root with Vertucci's type IV canal configuration^26^ and type V isthmus classification according to Hsu and Kim^9^ (1997), defined as the presence of complete communication between the two mesial canals. According to this classification, 30 mandibular molars were selected and randomly divided into two groups (n = 15).

Finally, the total volume of each root canal system was calculated by using the Ctan v. 1.12 software (Bruker-microCT), and the Mann-Whitney test was performed to confirm the even distribution of canal volumes between the two groups (P>0.05).

#### Sample preparation

The tooth crowns were removed at the cementoenamel junction level by using a 0.3 mm thick diamond disk fixed in an Isomet saw (Isomet, Buehler, Lake Bluff, Illinois, USA). The apical patency was determined by inserting a size 10 K-file (Dentsply Maillefer, Ballaigues, Switzerland) until it reached the apical foramen. Then working length (WL) was established at 1 mm short of the apical foramen.

A single operator prepared all the root canals using the Mtwo rotary instrumentation system (VDW, Munich, Germany) until a 35.04 diameter was obtained. Root canals were irrigated with 2.5 mL of 2.5% sodium hypochlorite (NaOCl) after each instrument by using a disposable syringe and a 27-G NaviTip needle (Ultradent, South Jordan, UT). On conclusion of root canal instrumentation, three applications of 2.5 mL of 2.5% NaOCl and 17% EDTA were made by means of passive ultrasonic activation (PUI) for 20 s each, to achieve improved isthmus cleaning^24^. Then, the root canals were finally irrigated with 5 mL of saline solution and dried with paper points.

#### Obturation of the specimens

The AH Plus sealer (Dentsply Maillefer, Ballaigues, Switzerland) was manipulated in accordance with the manufacturer's specifications. Rhodamine-B dye (Sigma-Aldrich, St Louis, USA) at 0.1% concentration was added to promote sealer fluorescence to enable viewing by confocal laser scanning microscopy (CLSM), as in previous studies^7^. Equal portions of approximately 0.1012 g of paste A and B were weighed in an analytical precision balance AR2140 (Ohaus Corporation, Shanghai, China) for the obturation of each specimen.

The sealer was inserted into the root canals with a Lentulo spiral #35 at a low speed of 200 rpm, by using the VDW Silver Reciproc motor (VDW, Munich, Germany) in rotary function. After sealer insertion in Group 1 specimens (n=15), the sealer was ultrasonically agitated (UA) for 20 s in each mesial canal, 2 mm short of the WL, in the bucco-lingual direction, by using an Irrisonic tip (Helse, São Paulo, SP, Brazil). For the purpose of UA, an ultrasonic device (Jet Sonic; Gnatus, SP, Brazil) was used at 20% of the power scale. In Group 2 (n = 15), no ultrasound activation (NUA) was applied after sealer insertion.

Subsequently, a 35.04 gutta-percha cone (VDW, Munich, Germany) was inserted into each canal up to the WL, and the material was seared off and compacted with a heated plugger 1 mm below the canal orifice. The excess sealer was removed and the coronal portion was sealed with a provisional filling material (Coltosol, Coltene, Altstatten, Switzerland). All specimens were then stored at 37°C in 100% humidity for 7 days to allow the sealers to set completely.

### Analyses of unfilled area, gaps at the sealer/dentine interface and sealer penetration

The specimens were horizontally sectioned at 2, 4 and 6 mm from the apex with a 0.3 mm Isomet saw (Buehler, Lake Bluff, IL) at 200 rpm, under continuous water cooling to prevent frictional heat, resulting in a total of 90 slices. All the slices were polished with a disc polishing machine under continuous water-cooling (Arotec, Cotia, São Paulo, Brazil) to produce a highly reflective surface.

A high-resolution stereomicroscope (Stemi 2000C; Carl Zeiss, Jena, Germany) was used at 8× magnification to calculate the percentage of unfilled areas. By means of Axiovision software (Carl Zeiss), the corresponding areas of the canals and isthmuses were divided and analysed separately. The total area of the canals and isthmuses of each cross section and the visible unfilled areas were measured. The percentages of unfilled areas in relation to the total area of the canal and isthmus were then calculated.

After evaluation by stereomicroscopy, all the samples were observed under an inverted Leica TCS-SPE confocal laser-scanning microscope using a 10× objective (Leica Microsystems GmbH, Mannheim, Germany) at 10 μm below the dentine surface. The absorption and emission wavelengths for rhodamine-B were set to 540 and 590 nm. All images were recorded using the fluorescent mode to a size of 1024×1024 pixels. Adaptation at the sealer/dentine interface and sealer penetration into the dentinal tubules of the canals and isthmuses were evaluated based on the methods used in other studies^13^ ^,^ ^14^ and use of Image J V1.46r software (National Institutes of Health, Bethesda, MD, USA). The canals and isthmuses were analysed separately. The perimeter of the canal walls was obtained; the areas in segments where the sealer penetrated into the dentinal tubules, and where gaps appeared at the sealer/dentine interface were obtained and converted into percentages. The same procedure was performed for the isthmus, measuring the mesial and distal walls only.

A single operator, blinded to the sample group, performed all the measurements, and the measurements were repeated twice to ensure reproducibility.

### Intradentinal antimicrobial activity

#### Sample Preparation

The crowns of thirty bovine incisor teeth and 2 apical mm were removed; the samples were standardized to 6 mm lengths by using an Isomet saw (Isomet 1000, Buehler Ltd, Lake Bluff, IL, USA) with a diamond disk at 250 rpm, under irrigation. The root canals were prepared using K files (Dentsply) up to #80. Teeth with a wider root canal were discarded. The inorganic part of the smear layer was removed with 17% ethylenediaminetetraacetic acid (EDTA) (Biodinâmica Química e Farmacêutica, Ibiporã, PR, Brazil) for 5 min, and the canals were washed with deionized water. Two layers of red nail polish (L'Oréal Colorama, Rio de Janeiro, RJ, Brazil) were used to cover the external surface of the samples, and dried for 24 h, before being autoclaved at 121°C.

#### Contamination protocol

**Figure 1:**
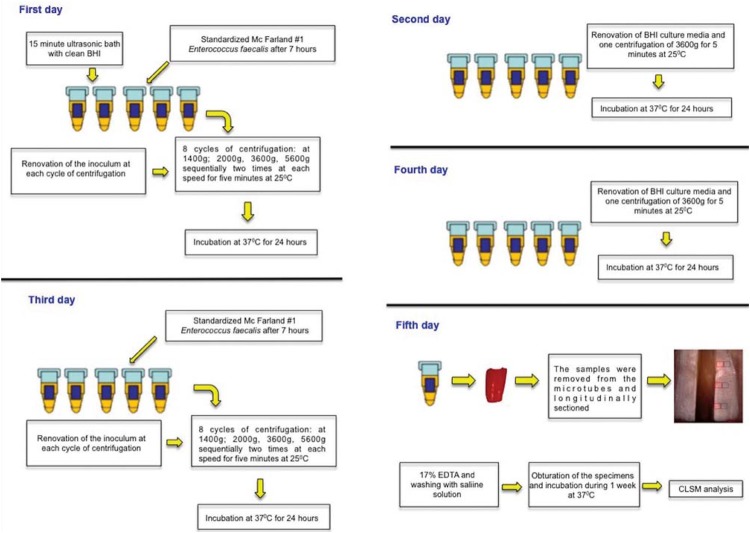
Flowchart of the contamination protocol

*E. faecalis* (ATCC 29212) was reactivated in Brain Heart Infusion broth (BHI, Difco, Kansas City, MO, USA) and maintained at 37°C for 24 h. The bacterial culture was transferred to another BHI flask and incubated for further 24 h in order to achieve exponential growth. This culture was adjusted to McFarland standard No. 1 (3×10^8^ CFU/mL) with a spectrophotometer (Bel Photonics do Brasil Ltda, Osasco, SP, Brazil). The contamination model, proposed by Andrade, et al.^1^ (2016), proceeded as follows (flowchart in Figure 1).

#### Sample Obturation

After the contamination procedure, the samples were removed from the BHI broth and dried with sterile paper points size 80 (Dentsply Maillefer, Tulsa, USA). The AH Plus sealer was manipulated on a sterile plate in accordance with the manufacturer's instructions. The sealer was placed in each root canal by using a K-File size 70 (Dentsply Maillefer, Tulsa, USA). Then the specimens were divided into 2 groups (n=10) according to ultrasonic activation of the sealer: ultrasonically agitated (G1) and non-ultrasonically agitated (G2) groups. In Group G1 activation was performed with an Irrisonic file (Helse, São Paulo, Brasil) adapted to an ultrasonic device (Jet-Sonic Four Plus; Gnatus, Ribeirão Preto, SP, Brazil) in “endo” mode (20% power). Because the ultrasonic oscillation occurs in a single plane, the file was activated for 20 seconds in the buccolingual direction as a standardization procedure. After this, an 80.02 guttapercha cone (Dentsply Maillefer) was inserted into the root canal to complete obturation and the excess was removed with a size 15 surgical blade. Group G2 was obturated in the same manner as G1, however, without previous ultrasonic activation. A third Group (G3) (n=10) did not receive any treatment and served as positive control. Afterwards, the specimens were inserted into sterilized microtubules and incubated at 37°C for one week.

### Confocal laser scanning microscopy (CLSM) analysis

After the above-mentioned time interval, the specimens were longitudinally sectioned by using an Isomet saw with a diamond disc, cooled with sterilized saline solution. The smear layer produced by this sectioning was removed with 2.5 mL of 17% EDTA for 3 min, and the samples were washed with sterilized saline solution. The halves of the dentinal tubes were stained with 30 μL of LIVE/DEAD^®^ *Bac*LightTM Bacterial Viability stain (Molecular Probes, Eugene, OR, USA) for 20 min. This kit contains SYTO 9^®^ dye, which stains live bacteria with a green pigment, and propidium iodine dye, which stains dead bacteria with a red pigment, thus enabling viable bacteria to be easily identified. The samples were examined by two blinded calibrated examiners with an inverted Leica TCS-SPE confocal microscope (Leica Microsystems GmbH, Mannheim, Baden-Württemberg, Germany) by using a 40X magnification oil lens. The images were obtained with 1 μm step size, in a 1024×1024 pixel format. For each specimen, 3 superficial (close to the root canal surface) and 3 deep (close to the external root surface) images were acquired and fragmented using the Leica Application Suite-Advanced Fluorescence software (LAS AF, Leica, Mannheim, Baden-Württemberg, Germany). For an objective analysis, the CLSM images were converted into “tiff” format by the LAS AF software. These images were exported to the bioImageL TM v21 software in order to quantify the percentage of live (green fluorescence) and dead (red fluorescence) bacteria.

### Statistical analyses

Because of the absence of normal distribution, observed by using the Shapiro-Wilk test, the Mann-Whitney test was used to analyse the influence of ultrasonic activation by means of stereomicroscopy and CLSM analysis. Bacterial viability data were evaluated statistically with the Kruskal-Wallis and Dunn tests. The Prism 6.0 software (GraphPad Software Inc., La Jolla, USA) was used as the analytical tool, and the level of significance was set at 5%.

## Results

### Root canal/isthmus filling quality

**Figure 2:**
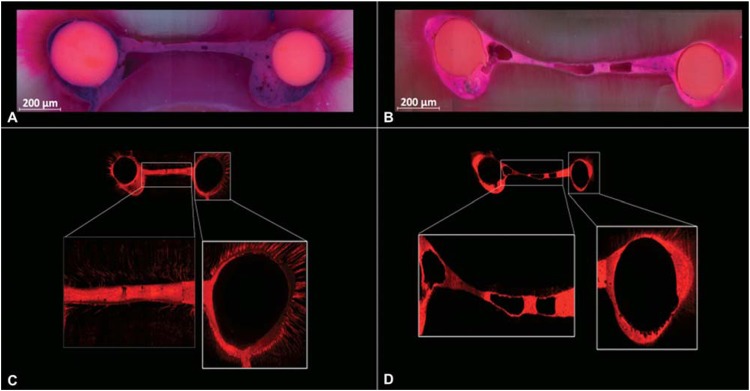
Representative stereomicroscopic (A and B) and confocal images (C and D) of filled mesial roots of mandibular molars at 4 mm from the apex. (A) Satisfactory filling without gaps in the canal and isthmus in the UA group. (B) Gaps inside the canal and isthmus in the NUA group. (C) A significant sealer penetration into the dentinal tubules of the canal and isthmus is observed in the UA group, contrary to the NUA group (D) where fewer dentinal tubules are filled with sealer

The median and the range of percentages of unfilled areas obtained from the stereomicroscopy analysis are shown in Table 1. In all sections, ultrasonic activation of the sealer significantly reduced the presence of unfilled areas in the canal and isthmus areas (p<0.05) (Figure 2A, B).

**Table 1:** Median, minimum and maximum values in percentage (%) of unfilled areas of the canal and isthmus

Canal			Isthmus		
Unfilled areas 2 mm (%)	Unfilled areas 4 mm (%)	Unfilled areas 6 mm (%)	Unfilled areas 2 mm (%)	Unfilled areas 4 mm (%)	Unfilled areas 6 mm (%)
0.0 (0.0 – 4.89)^a^	0.0 (0.0 – 3.28)^a^	0.0 (0.0 – 4.49)^a^	0.0 (0.0 – 11.98)^a^	0.0 (0.0 – 15.66)^a^	0.0 (0.0 – 11.88)^a^
0.11 (0.0 – 13.15)^b^	1.57 (0.0 – 13.65)^b^	2.33 (0.0 – 5.80)^b^	5.46 (0.0 – 53.27)^b^	14.38 (0.0 – 45.35)^b^	12.03 (0.0 – 66.02)^b^

A different letter in each column represents statistical differences between the ultrasonically agitated (UA) and nonultrasonically agitated (NUA) groups (p<0.05)

Results of the CLSM examination are presented as median and ranges in Table 2. Overall, ultrasonic activation of the sealer showed a significant improvement in sealer penetration into the dentinal tubules in both canals and isthmuses (p<0.05) (Figure 2C, D). Gaps at the sealer/dentine interface were significantly reduced at 2 and 6 mm in the isthmus area and at 6 mm only in the canals when the sealer was ultrasonically agitated (p<0.05). At 4 mm, there was no statistically significant difference between the groups (p>0.05).

**Table 2:** Median, minimum and maximum of gaps and sealer penetration segments (SP) in percentages (%) of the canal and isthmus

	Canal gap 2 mm (%)	Canal gap 4 mm (%)	Canal gap 6 mm (%)	Isthmus gap 2 mm (%)	Isthmus gap 4 mm (%)	Isthmus gap 6 mm (%)	Canal SP 2 mm (%)	Canal SP 4 mm (%)	Canal SP 6 mm (%)	Isthmus SP 2 mm (%)	Isthmus SP 4 mm (%)	Isthmus SP 6 mm (%)
UA	0.0 (0.0 - 9.49)^a^	0.0 (0.0 - 9.96)^a^	4.18 (0.0 - 16.68)^a^	0.0 (0.0 - 9.31)^a^	0.0 (0.0 - 32.63)^a^	0.0 (0.0 - 40.06)^a^	83.16 (76.91 - 100.0)^a^	89.03 (67.23 - 100.0)^a^	90.88 (58.58 - 100.0)^a^	87.05 (6.0 - 100.0)^a^	84.36 (24.33 - 100.0)^a^	76.25 (24.4 - 100.0)^a^
NUA	4.40 (0.0 - 20.46)^a^	6.43 (0.0 - 23.03)^a^	10.74 (0.0 - 28.07)^b^	0.0 (0.0 - 52.55)^b^	0.0 (0.0 - 30.69)^a^	2.50 (0.0 - 34.43)^b^	62.73 (35.14 - 100.0)^b^	74.77 (38.80 - 100.0)^b^	71.47 (19.12 - 100.0)^b^	0.0 (0.0 - 82.11)^b^	4.89 (0.0 - 100.0)^b^	36.79 (0.0 - 76.22)^b^

A different letter in each column represents statistical differences between the ultrasonically agitated (UA) and nonultrasonically agitated (NUA) groups (p<0.05)

### Intradentinal antimicrobial activity

**Figure 3:**
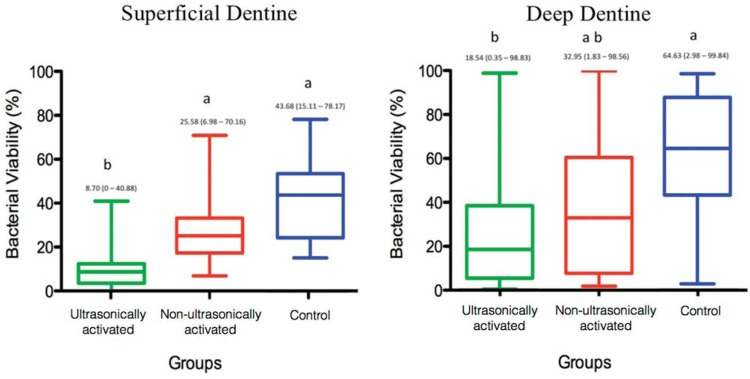
Graph representing the median, minimum and maximum range of the percentage of bacterial viability on the superficial dentine (A) and deep dentine (B): ultrasonically activated (G1), non-ultrasonically activated (G2) and control group (G3). Different letters represent statistical differences between the groups

The percentages of bacterial viability in superficial and deep dentine are shown in Figure 3. In superficial dentine, ultrasonic activation significantly reduced the bacterial viability when compared with the other groups (p<0.05). As regards deep dentine, there was a significant reduction in bacterial viability only when the ultrasonically agitated group (G1) was compared with Control group (G3) (p<0.05).

## Discussion

Ultrasonic activation of the endodontic sealer significantly improved the filling quality (canal and isthmus), adaptation of sealer/dentine interface and intratubular penetration in the UA group. The antimicrobial activity was also enhanced by UA in superficial dentine. Therefore, our entire null hypothesis was rejected. The favourable results obtained have a significant importance, if we consider previous studies^3^ ^,^ ^25^ ^,^ ^27^ that showed deficiency in properly filling and disinfecting these complex areas was capable of leaving tissue remnants, bacteria and debris, increasing the risk of post-treatment apical periodontitis.

In the present study, PUI for 1 min *per* canal was performed in both groups to achieve an improved isthmus cleaning^8^ and the sealer was observed to have the ability to flow into cleaned isthmuses in both groups. However, a low quantity of sealer was observed inside the isthmus in the NUA group, leading to greater presence of unfilled areas in all the cross sections, irrespective of the isthmus width or length (Figure 2B).

Furthermore, to analyse the influence of ultrasonic activation of the sealer and quantify only the sealer component inside the isthmus, the single cone technique was used in root canal fillings. This was done to enable proper differentiation between gutta-percha and sealer, because when thermoplasticised techniques are used, a single obturation mass is produced, making it difficult to differentiate between the two materials separately. Indeed, Marciano, et al.^13^ (2011) reported that the presence of isthmuses increased the incidence of unfilled areas, even when using thermoplasticised techniques. Moreover, the single cone technique allowed the insertion of a standard amount of gutta-percha with reduced compaction forces when compared with other techniques that could push the sealer and gutta-percha into the isthmus, thereby interfering with the main study objective. The above-mentioned factors, previous anatomical pairing, and the similar sealer volume inserted into each specimen may have provided more reliable results. Therefore, the results found in the UA group possibly occurred exclusively as a result of the ultrasonic activation of the sealer.

The improvement in the amount of sealer that entered inside the isthmus in the UA group was probably caused by the transmission of acoustic microstreaming energy produced by the ultrasonic tip, which could have forced the sealer into these areas. This effect regularly occurs in the irrigating solution while performing passive ultrasonic irrigation^8^ ^,^ ^24^.

Although the results of the confocal microscopic analysis of both the groups showed gaps at the sealer/dentine interface, ultrasonic activation of the sealer improved this aspect by significantly reducing the gaps to minimum values in some canal cross sections. Furthermore, higher percentages of intratubular sealer penetration were observed in the canal and isthmus region of the UA group at all the levels evaluated. Previous studies presented similar results using single rooted teeth^7^ ^,^ ^16^. Macedo, et al.^12^ (2014) showed that the ultrasonic activation increased the temperature of irrigants. The same phenomenon can occur during ultrasonic activation of the sealer, which could promote higher flowability and reduce the film thickness, favouring its penetration into dentinal tubules. The medians of sealer penetration of the UA group in all sections were above 76% when compared with the NUA group, in which penetration values were below 39%. This result is noteworthy if we consider the antibacterial properties of root canal sealers within the dentinal tubules^29^.

This study presented a method for intratubular contamination of bovine dentine proposed by Andrade, et al.^1^ (2016). Bovine teeth are commonly used as an experimental substitute for human teeth because they are easily available^21^. Compared with human dentine, bovine dentine has a higher concentration of dentine tubules *per* square millimeter, however, this difference is small. On average, the diameter of dentine tubules of bovine teeth is larger than that of human teeth, but this difference is not statistically significant. The percentage of intertubular dentine in bovine teeth is the same as it is in human teeth^21^.

**Figure 4:**
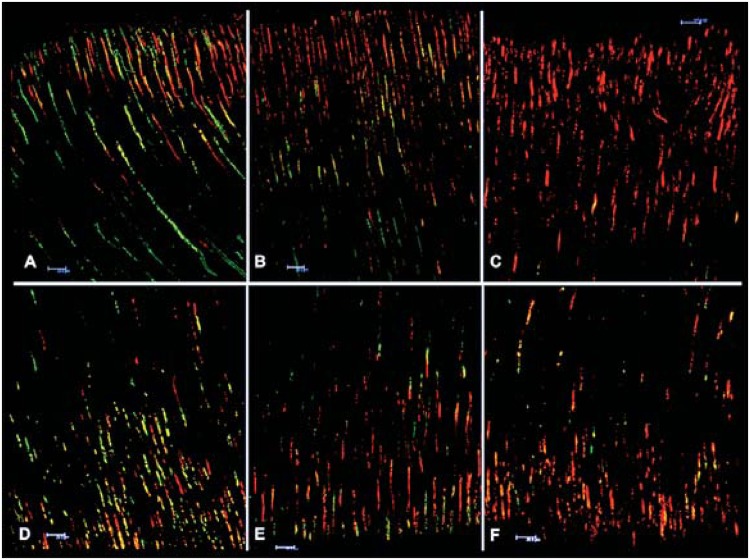
Representative images of bacterial viability in superficial (A, B and C) and deep (D, E and F) dentine obtained by confocal laser scanning: A, D (control group), B, E (without ultrasonic activation of the sealer) and C, F (with ultrasonic activation). Red colour denotes dead bacteria and green denotes live bacteria

Vera, et al.^25^ (2012) showed that microorganisms remain inside the dentinal tubules and survive after biomechanical preparation and intracanal dressing. Thus, the sealer penetration into the dentinal tubules plays an important role during root canal filling because of the antimicrobial action of these materials and their action of entombing residual microorganism^10^ ^,^ ^15^. Our results showed that ultrasonic activation of the sealer promoted a significant reduction in *E. faecalis* viability in superficial dentine when compared with the other groups (Figure 4A, B and C). As regards deep dentine, there was statistically significant reduction in *E. faecalis* viability only in the ultrasonically agitated group in comparison with the Control group (Figure 4C, D and F). The AH Plus sealer has antimicrobial action against *Enterococcus faecalis* ^18^ ^,^ ^28^. The antimicrobial properties of epoxy resin-based sealers may be related to the bisphenol-A diglycidyl^23^ or small quantities of formaldehyde^11^ released into the dentinal tubules during the polymerisation process.

The possible explanation of the improvement of the antimicrobial action of the AH Plus when activated by ultrasonic tip is due to the transmission of acoustic microstreaming energy produced, which increases the sealer penetration into the dentine tubules^7^ ^,^ ^16^. Thus, the greater sealer penetration may act as a physical barrier and may entomb residual microorganisms, thereby separating them from nutrient sources^17^. In addition, the greater contact between the sealer and microorganisms could favour the antimicrobial action of the AH Plus, consequently reducing the microorganism viability.

## Conclusion

The ultrasonic activation of the AH Plus sealer promoted a better quality of root canal filling and increased the intratubular penetration of sealer, especially in the isthmus area. Additionally, ultrasonic activation of the sealer increased the intradentinal antimicrobial action against *Enterococcus faecalis,* mainly in the superficial dentine of the root canal.
